# Sympathetic Vomiting of Pregnancy

**Published:** 1885-08

**Authors:** W. T. Baird, R. G. Williams

**Affiliations:** Albany, Texas; Whitney, Texas


### PERSISTENT SYMPATHETIC VOMITING OF PREGNANCY.

TWO CASES.

*One reported by W. T. Baird, M. D., Albany, Texas; and one by R. G. Williams, M. D., Whitney, Texas.*

For Daniel's Medical Journal.

### CASE NO. 1.

Dr. Baird, says:

I recently treated a young married lady for obstinate vomiting due to pregnancy; and as the result was so satisfactory,—whereas the usual treatment of these cases is generally so unavailing, and so trying both to patient and physician,—I am induced to give history and treatment of the case.

Mrs. A. R., aged 19, married in September last, and pregnant now three months. She had always been healthy, though of a rather delicate constitution. She commenced vomiting two months ago, (present date, July 5), and continued till she became quite emaciated; then restlessness set in, and day nor night she could not sleep, only for a few minutes at a time. On swallowing anything whatever, either food or drink, vomiting was provoked, and she suffered constantly with a distressing nausea, even while the stomach was empty, accompanied by a most terrible heart-burn constantly; great jactitation and rolling about on the bed and wildly tossing her arms. (These were not symptoms of hysteria—there was no such complication.) Her temperature was normal, and her pulse ranging from 160 to 180 per minute, weak and easily compressed. A large herpetic ulcer occupied each angle of her mouth. The walls of the stomach were so greatly thickened by conjestion of its vascular tissue that it presented the appearance of a large pulsating tumor which could be plainly seen beating, as she lay on her back. The abdominal walls were tense and rigid; bladder containing nearly a pint of light colored urine, although she said she had voided her urine a few minutes previous to my arrival. Uterine hemorrhage had set in the night before, and she was now threatened with abortion. The tongue was coated with a thick yellowish fur, tips and edges intensely red and prsented the appearance of a piece of raw beef; surface dry and harsh. The only pain complained of was that in her stomach, which was constant and excessive.

She had been under the treatment of my esteemed colleague Dr. Powell, who had been called away a long distance, hence my services were required. The Doctor had given her carbolic acid, and a cathartic, also some other remedies, and had brought over his electrical apparatus, but I believe did not use it.

Having, from experience in these cases, little faith in medication, by the mouth especially, seeing the stomach is in such condition that all absorption must be arrested, I at once resorted to the use of the electric current. At first I employed a current from the primary, with 1st and 2nd induction coils,—positive in the vagina, electrode insulated to near its tip, which point was brought in contact with the os uteri; then, using my hand for the negative, I passed it gently over the abdominal region and over the spine, for about 20 minutes, with the current as strong as she could bear with comfort. Then, using the same current, I applied the positive with a sponge electrode over the region of the stomach, and the negative on the spine, opposite, also over the right hypochondriac region, for abont 10 or 15 minutes. The position of the poles were varied,—at times a current was sent down the spine, negative to os coxcygis and positive to cervical region, for a few minutes.

This treatment, with the omission of application to the os, was instituted twice a day for four days. The first application arrested the uterine hemorrhage while the current was being applied, and it only recurred once, and then only for a few minutes. The jactitation subsided at once and did not recur. The first night, she slept better; second day, her pulse was 140 to 160, other symptoms unchanged. The third day, pulse had fallen to 120 to 140, while the pain in the stomach had become less intense; tongue cleaning off and losing the fiery redness; bowels softer, stomach diminishing in size, urine high colored (but containing no bile) and voided without difficulty. The fourth day, all symptoms improving; pulse 120. Ate occasionally on the fourth day without experiencing either nausea, retching or vomiting, and although relieved of the distressing symptoms, she was too feeble and too much prostrated from the exhausting effects of emesis, and the privation of food and drink, to sit up in bed, and when raised, experienced vertigo. On the fifth day,—by which time the seances or applications of the electric current had been reduced to one only,—her pulse was fuller and stronger, and she ate well her accustomed food without a return of the nausea and vomiting, and was dismissed feeling, as she said, 'perfectly well,' and being able to sleep as in health. She sat up a few moments on the fifth day. When I called again, on the sixth day from my first visit, I found there had been slight return of the nausea before breakfast, but not afterwards, and at this visit I found her perfectly comfortable and able to eat her three meals a day without discomfort or without a return of the vomiting; meantime the abdomen had softened and the bowels had acted spontaneously, whereas enemas had been used previously; tongue cleaned off and presenting almost a normal healthy appearance. I had no fears for her safety now, and dismissed the case.

---

CASE NO. 2.

DR. R. G. WILLIAMS, reports:

Mrs. K. M., Colorado, Texas, aged 23, married May 1st, 1884; general health good, usual weight 130 pounds. Early in October of same year, she aborted at sixth week. By the following December, her general health was fully restored, and continued excellent until April 1st, 1885, when she again conceived. Nausea was one of the earliest and most persistent symptom; for the first two weeks in April, vomiting came on only in the afternoons, but by the 15th inst, it was as troublesome in the mornings. About this time, Dr. C. was called in. By May 1st, this vomiting was more or less constant through the night, as well as the day. Dr. T. was now called in some time later, but failing to give the desired relief, her husband desired my services. I reached her bedside on the morning of June 11th.

At this date, she was greatly emaciated, restless, and unable to sleep more than two out of every twenty-four hours. Bowels costive, moving only by daily enemas ; mentally depressed, extremely nervous, complete loss of appetite, and vomiting almost constantly.

Believing the former attendant physicians had about exhausted all medical treatment, I determined to withhold all medicines and treat my case wholly with electricity. For this purpose, I used the Faradic battery, primary, with 1st and 2nd induction coils, positive electrode to cervical vertebra, negative to epigastrium, length of application 20 minutes ; then with stronger current passed the current down the spine, labial application, time 5 minutes ; then placed positive electrode over coccyx, using negative over lower extremeties, 3 minutes. In this way, electricity was used, twice on the 11th, three times each on the 12th and 13th, when my battery failed to perform. No benefit whatever was perceptible until after the 5th application, when my case slept well all night, awakening on the morning of the 14th, calling for food. Food and fluids were now given liberally, and retained.

On this day she determined to return with me to my home for further electrical treatment, and though the distance was 250 miles, the journey was made in twenty-four hours, and without once vomiting.

On the 15th, electrical treatment was again instituted, and continued morning and night with steady improvement, until the 20th, when mastitis supervened.

This was at first treated by the application of the P. A. Harris bandage, but from faulty arrangement, it failed to give the relief expected, and on the morning of the 21st, both glands being greatly enlarged and extremely painful, preventing sleep the previous night, I began the electrical treatment directed especially to the breasts, applying the positive electrode to gland with the negative to upper dorsal vertebra, then passed the current directly through the mamma from side to side.

After the fourth application I heard no further complaint, and ceased all treatment, except general tonic electrical treatment once per day, until the 6th of July, when she returned home.

On the day of her departure from home she weighed 110

pounds; on the day of her return she weighed 123 pounds. All of the other symptoms had proportionately improved. The bowels were moving normally; the appetite was splendid. She rested well each night, and slept an hour or two each day. She was now cheerful and buoyant, and began to show roses on each cheek.

July 24.—Have just received a letter from my case, stating that she is doing finely.

